# Malaria-Associated Factors among Pregnant Women in Guinea

**DOI:** 10.1155/2019/3925094

**Published:** 2019-11-15

**Authors:** Almamy Amara Touré, Abdoulaye Doumbouya, Abdourahamane Diallo, Gaspard Loua, Abdourahim Cissé, Sidikiba Sidibé, Abdoul Habib Beavogui

**Affiliations:** ^1^Centre National de Formation et de Recherche en Santé Rurale de Maferinyah, Forecariah, Guinea; ^2^Université Gamal Abdel Nasser de Conakry, Conakry, Guinea; ^3^Centre Hôspitalo-Universitaire Ignace Deen, Service de Gynecologie, Conakry, Guinea; ^4^Johns Hopkins Program for International Education in Gynaecology and Obstetrics (JHPIEGHO-Guinée), Conakry, Guinea; ^5^Sightsavers, Conakry, Guinea

## Abstract

**Introduction:**

Malaria is the leading cause of consultation in Guinea health facilities. During pregnancy, it remains a major health concern causing considerable risks for mother, fetus, and newborn. However, little is known about the epidemiology of malaria among pregnant women in Guinea. We aimed to provide information on malaria-associated factors in parturients.

**Methods:**

It was a cross-sectional survey in two regional hospitals and two district hospitals. 1000 parturients and their newborns were surveyed. All patients were interviewed, and thick and thin blood smears were examined. To determine the predictive factors of malaria in parturients, the Classification and Regression Tree (CART) was first performed by using peripheral and placental malaria as dependent variables and sociodemographic and antenatal characteristics as independent variables; then, explanatory profile variables or clusters from these trees were included in the logistic regression models.

**Results:**

We found 157 (15.8%) and 148 (14.8%) cases of peripheral and placental malaria, respectively. The regular use of long-lasting insecticide-treated nets (LLINs) before delivery was 53.8%, and only 35.5% used sulfadoxine-pyrimethamine doses ≥3. Factors significantly associated with malaria were as follows: women from Forécariah and Guéckédou who did not regularly use LLINs and accomplished less than four antenatal care visits (ANC <4) and primigravid and paucigravid women who did not regularly use LLINs. Similarly, the odds of having malaria infection were significantly higher among women who had not regularly used LLINs and among primigravid and paucigravid women who had regularly used LLINs compared to multigravida women who had regularly used LLINs.

**Conclusion:**

This study showed that pregnant women remain particularly vulnerable to malaria; therefore, strengthening antenatal care visit strategies by emphasizing on promoting the use of LLINs and sulfadoxine-pyrimethamine, sexual education about early pregnancies, and family or community support during first pregnancies might be helpful.

## 1. Introduction

Malaria infection during pregnancy is a major health concern with considerable risks for mother, fetus, and newborn [1]. According to the 2007 forecast, 25.6 million pregnancies occurred in areas at risk of malaria [2]. A systematic review of studies conducted between 1985 and 2000 indicated that the prevalence of malaria infection during pregnancy ranged from about 10% to 65% in the contexts where these associations were observed [3]. Impact of malaria on the outcome of pregnancy could result in low birth weight (LBW), stunting, or maternal anemia [4–8]. Prevention of malaria during pregnancy is vital and allows substantial reductions in neonatal mortality and LBW [9]. According to Tailor et al., extrapolating the prevalence of malaria to the population at risk in the Democratic Republic of Congo suggests that 1.015 million births are affected each year by *Plasmodium falciparum* infection; the authors had estimated that the application of preventive measures could have prevent 549,000 episodes of malaria associated with pregnancy and 47,000 cases of LBW [10]. This endemic context of malaria in many countries has led the World Health Organization (WHO) to recommend since 2004 till now, the implementation of a set of measures, including the promotion and use of long-lasting insecticide-treated nets (LLINs), intermittent preventive therapy in pregnancy (IPTp) with sulfadoxine-pyrimethamine (SP), and adequate management of cases of malaria in pregnant women in endemic countries [1].

Malaria is the leading cause of consultation in health facilities in Guinea; according to Multiple Indicator Cluster Survey (MICS), the prevalence ranged between 8 and 30% depending on the region [11]. This proportion down from previous years is the result of malaria control initiatives, which have escalated recently, mainly among pregnant women and children under five, with chemoprevention, free mass distribution of LLINs in 2013 and 2016, free treatment of malaria cases, and the promote of health community care by the National Malaria Control Program (unpublished data). Despite all these measures, the percentage of pregnant women who received optimal doses of SP remained relatively low, 24% in 2012 [12] and 30% in 2016 [11]. As for the use of LLINs, only 54% of women aged 15–49 used it the night preceding the MICS [11]. In order to evaluate the impact of preventive measures, demographic and health survey was carried out in children under five and hardly in pregnant women. And therefore, with lack of information on the prevalence of malaria in the latter group, progress achieved is difficult to track, especially with low SP and LLINs coverage. This study aimed to identify the factors associated with malaria in parturients in the context of implementation of multiple preventive measures and after Ebola virus disease outbreak.

## 2. Materials and Methods

### 2.1. Characteristics of the Study Sites

This study was carried out in four hospitals, two of which were considered as district hospitals (first-level reference for health centers) and the other two as regional hospitals (second-level reference for health centers). Forécariah district hospital is located about 100 km from the capital Conakry; the district has 242,942 inhabitants, with rainfall for 6 months in a year and vegetation consisting of mangrove forests. Guéckédou district hospital is located about 600 km from the capital; the district has about 290,611 inhabitants, with rainfall for 10 months and vegetation consisting of dense forest. Kankan regional hospital is located about 700 km from the capital, with an estimated population of 2,097,257 inhabitants, with an estimated rainfall for 6 months but lower than that of Forécariah and vegetation made of grassy savannah. N'Zérékoré regional hospital is located about 950 km from the capital; the district has about 1,686,799 inhabitants with a rainfall for 10 months in a year and vegetation consisting of dense forest. These data were obtained from unpublished studies.

### 2.2. Type, Period, and Study Population

This study was a cross-sectional survey of 1,000 parturients and their newborns. The sample was obtained based on the prevalence of malaria among pregnant women in Burkina Faso given the lack of information in Guinea [13], with *n*=*Z* ^2*∗*^ (*P* *∗* *Q*)/*i* ^2^, where *n* is the desired sample size; therefore, prevalence (*P*) = 18%; *q* = 1 − *p* (expected prevalence in the population); *p* = 88%. *Z* level of confidence according to the reduced normal centered law (for a 95% confidence level, *α* = 0.05, one has the *z* value of 1.96). By fixing *i* = 5% as the desired precision on the sample size. The minimum sample size was 226 parturients, taking into account the loss of biological material at 10%, this size was increased by 227/0.90 = 252 parturients and was finally 250 by district hospital. The study ran from May to September 2017, corresponding to the rainy season in Guinea.

### 2.3. Training

Four medical school students working on their dissertations were trained at all stages of the investigation. A pretest was organized prior to the start of the survey to ensure consistency of investigational tools.

### 2.4. Digital Puncture in Parturient and Newborns

After stinging the fingertip in parturient and the heel in newborn, about 5 *μ*l of blood (3–5 drops for the thick blood and 2 drops for the thin smears) was collected to make thick and thin blood smears which was dried and stained with a solution of Giemsa 10%. Peripheral malaria was diagnosed only by taking into consideration maternal test results.

### 2.5. Placental Sampling

After delivery, a piece of placental cotyledon was cut on the maternal side of the placenta and placed on the slide. With another slide, the piece of cotyledon was triturated to make thick and thin blood smears.

### 2.6. Examination of Slides

Thick and thin blood smears were examined by two certified microscopic biologists. A third microscopist intervened in cases of unconformity for more than 30% between the first 2 microscopists.

### 2.7. Ethic Statements

For the smooth running of this study, it required the approval (0018/DM/CPM/17) of the Faculty of Medicine, and authorizations were obtained from heads of the different health facilities where the study has been conducted. Written inform consent was obtained before the study from all participants after explaining them the purpose of the study.

### 2.8. Statistical Analysis

The data were first entered in Access 2013 and then exported to Excel in comma-separated value (CSV) format. Data were summarized by frequencies and percentages for the categorical variables; continuous variables were analyzed by the average with the standard deviation. In univariate logistic analysis, associations were determined between the dependent variables (peripheral malaria and placental malaria) and sociodemographic variables. To identify factors associated with malaria among parturients, we used two complementary approaches:

 
*Step 1* : The classification supervised with the CART (Classification and Regression Tree) method to identify the variable profiles or clusters.
  (i) *Supervisers*. Peripheral malaria and placental malaria
  (ii) *Variable of Classification*. Age of the parturient, the regular use of the LLINs, preterm delivery, parity, gravidity, monogamic or polygamic regimen, irregular collected garbage, stagnant water around the house, source of water, lived time, characteristic of residence, level of education, profession of parturients, profession of head household, use of medicinal plant against malaria, the means of transport used to accomplish antenatal care visit (foot, taxi, and personal means), marital status, status of household, number of ANCs visits, distance from ANC health facility, and SP doses
 
*Step 2* : Logistic regression to quantify the risks (OR) associated with the profiles (clusters).
  (i) *Variable to Explain*. Peripheral malaria and placental malaria
  (ii) *Explanatory Variables*. The variable profiles obtained during the classification

The following variables were recoded before analysis: age (14–18, 19–35, and 36–45), ANC (<4 low and ≥4 normal), SP dose (<3 and ≥3), gestational (1 = primigravid, 2–3 = paucigravid, and >3 = multigravida) and parity (1 = primiparous, 2–3 = pauciparous, and >3 = multiparous).

The use of CART allows us to overcome the issues of multicollinearity; the overall significance of the model was tested by the likelihood ratio; the Pearson residual test was performed for the relevance of the model and the ROC curve was used to assess the quality of our model. All analyses were done using the *R* software (version 3.5.1). The statistical tests were performed at the risk threshold *α* = 5%. All the values of *p* < 0.05 were considered significant for the interpretation.

## 3. Results

The average age of women surveyed was 24.38 ± 6.2, and those aged from 19 to 35 accounted for 75.2% followed by the category of 14 to 18 (19.3%). Married women standed for 79.1% of our sample (additional file 1).

The age of pregnancy was at term (≥37 weeks) in 60.4% of cases. Eighty-two percent of parturients lived in the same district where they gave birth for more than six months. The majority of women (72.7%) lived in urban areas. Most of the women surveyed (51.8%) used the well as a source of water, followed by rainwater (30.6%). As for the level of education, 56.2% had no level, followed by the secondary level (23.4%) and the primary level (15.9%). Housewives made up 46.9% of the sample, 34.8% self-employed women, and 18.3% civilian servants. 49.2% of women lived in monogamic regimen and 31.2% in polygamic one (Table 1). Stagnant water and garbage were, respectively, present in 29.1% and 42%. 53.9% of women accomplished their ANC visits on foot, 27.3% by taxi, and 18.8% with their own means of transportation (additional file 1). Women who lived less than five kilometers from the health facility accounted for 87.7%. Living newborns accounted for 94.5% (additional file 1). The regular use of LLINs prior to delivery was at 53.8%. Only 30% accomplished at least four ANC visits. Primigravids accounted for 37.6% and paucigravids accounted for 33%, among which 0.7% were primiparous and 59.7% were pauciparous (additional file 1). Only 35.5% had received at least three doses of SP during pregnancy, and 40.3% received other antimalarial drugs (additional file 1). Finally, 31.2% of women used traditional medicines during pregnancy (additional file 1).

### 3.1. Risk Factors for Peripheral and Placental Malaria in Univariate Analysis

In univariate logistic analysis, the following variables increased the odds of having malaria: residence (Forécariah and Guéckédou), characteristic of residence (rural), source of water (well), head of household profession (farmer), means of transportation for ANC (foot), no regular use of LLINs, low ANC, and no use of medicinal plant (Table 1); while lived time (≥6 months), parturient profession (civil servant) and SP doses (≥3 doses) were negatively associated with malaria (Table 1).

### 3.2. Risk Factors for Women Who Had Both Peripheral and Placental Malaria (Additional File 3, Table 1)

12.2% of parturients had both peripheral and placental malaria. In addition to the results observed in Table 1, we found that pregnant women from N'zérokoré are 52% less likely to have malaria infection compared to those from Kankan (additional file 3, Table 1). Similarly, we computed risk for women who had only peripheral malaria; we found no association between dependant variable and risk factors (additional file 3, Table 2).

### 3.3. Factors Associated with Peripheral Malaria Using CART

**Figure 1:**
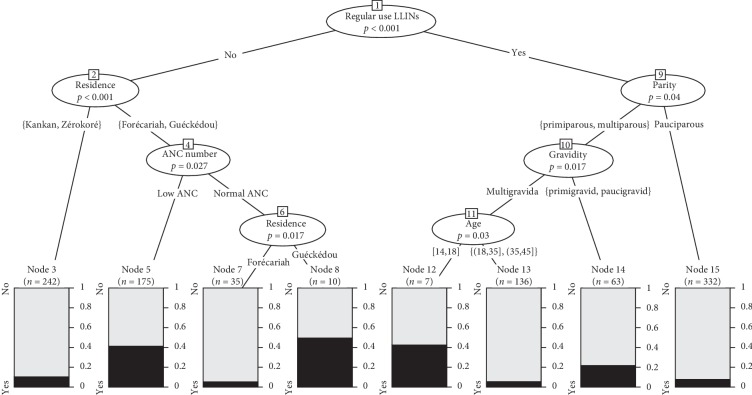
Associated factors with peripheral parasitaemia using CART. *Note*. Nodes correspond to the elements of clusters. The boxes represent the pregnant women screened for malaria, the grey ones stands for negative while the black ones positive.

Regular use of LLINs during pregnancy discriminates four main clusters at risk of malaria with peripheral parasitemia: two clusters for no regular use of LLINs (5 and 8) and two clusters (12 and 14) for the regular use of LLINs. Cluster 5: pregnant women who lived in Forécariah or Guéckédou with less than four ANC visits. Cluster 8: pregnant women who lived in Guéckédou with at least four ANC visits. Cluster 12: young women multigravida between 14 to 18 years old. Cluster 14: paucigravids and primigravids (Figure 1).

### 3.4. Predictive Factors of Malaria according to Peripheral Malaria (Table 2)

Forécariah and Guéckédou pregnant women who did not regularly use LLINs with less than four ANC visits were about 12 times more likely to be infected at delivery compared to women in Forécariah who completed at least four ANC visits. The odds of Guéckédou pregnant women who did not regularly use LLINs before delivery with at least four ANC visits were significantly higher compared to those of Forécariah pregnant women with at least four ANC visits associated to the irregular use of LLINs. Multigravida aged from 14 to 18 years old who regularly used LLINs were 12 times more likely to be infected at delivery compared to those who lived in Forécariah with at least four ANC visits and irregular use of LLINs. The odds of women who consistently used LLINs among primigravids and paucigravids were significantly higher compared to women who lived in Forécariah with at least four ANC visits and irregularly use of LLINs.

### 3.5. Factors Associated with Placental Malaria Using CART

**Figure 2:**
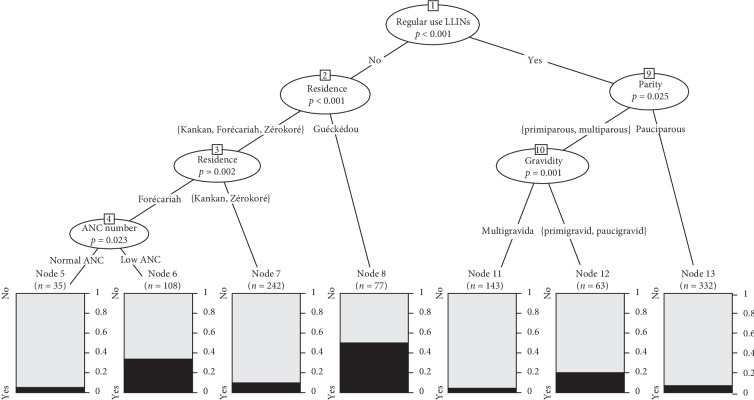
Associated factors with placental parasitemia using CART.

Regular use during pregnancy discriminates three main clusters at risk of malaria with placental parasitemia. Cluster 6. Women who lived in Forécariah with less than four ANC visits. Cluster 8. Women who had not regularly used LLINs and who lived in Guéckédou. Cluster 12. Primigravids and paucigravids who have regularly used the LLINs (Figure 2).

### 3.6. Predictive Factors of Malaria according to Placental Malaria 

The odds of Forécariah women who did not regularly use LLINs with ANC <4 were significantly higher compared to multigravida women who regularly used LLINs (OR = 10.12, 95% CI 4.54–25.84, *p* < 0.001). Guéckédou women who did not regularly use LLINs were 19 times more likely to be infected at delivery compared to multigravida women who regularly used LLINs (OR = 19.94, 95% CI 8.73–51.99, *p* < 0.001). The odds of women who consistently used LLINs among primigravids and paucigravids were significantly higher compared to multigravida women who regularly used LLINs (OR = 5.05, 95% CI 1.95–14.12, *p* < 0.001) (Table 3).

## 4. Discussion

After the implementation of multiple measures of malaria prevention in Guinea and the occurrence of the Ebola virus disease outbreak in 2014, we intended to conduct a cross-sectional survey in order to investigate factors associated with malaria in pregnant women in Guinea. We found that multiple factors are associated with malaria.

In univariate analysis, we noticed that young women are more likely to have placental parasitemia than women aged from 35 to 45 years old. This may be explained by their experiences in pregnancy management compared to young women who are 14 to 18 years old who lack experiences. Residence (Forécariah and Guéckédou) was also a predictor factor for both peripheral and placental parasitemia. Those sites are bordered districts of neighbouring countries (Sierra Leone and Liberia), which had affected during Ebola virus outbreak in 2014 and 2015. Pregnant women who lived more than six months in their place were more protected owing to apparently their adaptability. This study also showed that women who lived in rural settings were more exposed to malaria infection than those in urban. Source of water, profession of parturient, profession of head of household, and means of transportation for ANC are not directly related to malaria but may influence pregnant women health. Socioeconomic conditions could explain the lack of means to comply with the NMCP guidelines regarding malaria prevention. However, we did not find any statistical relation between malaria and women education. The interesting thing revealed in univariate analysis was the significant relation between peripheral malaria and medicinal plants. This seemed to be effective for women; however, at this stage, it is difficult to rely on this result to draw sound conclusion. Further investigation would be requisite in order to assess its protective effect. Efficacy and effectiveness of SP in pregnancy rely on evidence [9, 14–17]; more courses of SP provide more efficacy. The policy of intermittent preventive treatment of malaria in pregnancy with sulfadoxine-pyrimethamine in Guinea recommends delivering SP free of charge at each ANC attend. However, we observed that only 30% had done 4 ANCs or more. This low coverage can be explained by the failure to maintain the two following strategies: (1) advanced strategy: which consist to provide services to women in remote areas; (2) active research: which consist to actively seek out women who have made initial contact at the health centre.

We were also interested in computing predictive factors for women who had both peripheral and placental parasitemia to find out whether other factors are associated with malaria. Interestingly, we found that women from N'Zérokoré had less chance for having malaria compared to those from Kankan. In fact, many interventions from Non-Government Organizations and donors takes place in N'Zérokoré. Those interventions reinforced the existing measures of malaria prevention, including delivery of SP, ANC visits, and use of LLINs. Unlike the previous result, women who had only peripheral infection revealed no association with predictive factors. The sample size of these women (*n* = 36) might not be enough to detect associations. Moreover, most studies highlighted that placental parasitemia plays a key role in malaria during pregnancy [8, 18–22].

Furthermore, we also found that the use of LLINs remained below expectations (only 54% used it regularly before delivery) despite the repeated mass distribution campaigns. This study showed, on a large scale, its major predictive role in malaria transmission. Parturients from Forécariah and Guéckédou are the most affected; in addition, these women who did not use the nets regularly had inefficient ANC visits, which exposed them more to the risk of malaria. As previously discussed, ANCs allow to detect abnormalities during pregnancy. Uptake of its advantages can potentiate its use; its contribution in the prevention of malaria has been demonstrated in several studies [23–26]. It also emerged from this study that the majority of pregnant women had no level of education, which might at some extent limit both the use of LLINs and ANC visits. Beyond these ANC visits, it should be noted that even educated women could sometimes ignore the use of LLINs. This low use of LLINs could be explained by sociodemographic characteristics (low knowledge of its interest, environment of residence, allergies, choking, inappropriate form of LLINs, and so on) [27, 28]. The other aspect that influences the risk is the socioenvironmental context of these two districts (Forécariah and Guéckédou). In fact, these regions had been seriously affected by the Ebola virus disease outbreak, which has not only undermined local health systems but also led to behavioral changes regarding health facilities.

Young women (aged 14–18), multigravida, were another cluster susceptible to malaria infection. That gives rise to two major issues: (1) early marriage (physical and psychological immaturity) and (2) pregnancy outside marriage, more than half of this age group was not married (supplementary file 2). Teenagers accounted for a significant proportion of our sample (19%), which is in accordance with that reported by the MICS (31% of women aged from 15 to 19 had started their reproductive life) [11]. That situation is more worrisome because it mainly concerns teenagers in urban areas. Studies highlighted the negative impact of pregnancy on adolescence; Mombo-Ngoma et al. have shown that pregnancy in teenagers results in LBW babies and prematurity [29]. Our results also converge on other studies that also indicate negative associations between pregnancy and teens [30–32]. Primigravids and paucigravids who regularly used LLINs before delivery was the last cluster at risk. The first pregnancies are often at risk due to a decrease in immunity, which exposes pregnant women to infections [33–36]. The design of this study does not establish the causal association. Similarly, the use of regional and district hospitals that are reference structures is another limitation of the study. The 2016 MICS survey showed that the proportion of deliveries at home was 43% [11], which potentially leads to selection bias. Despite these limitations, the study is a reference in a context of scarcity of data.

## 5. Conclusion

This study showed that pregnant women remain particularly vulnerable to malaria. The measures undertaken by the National Malaria Control Program collide with the sociodemographic realities of these women, so regular use of LLINs and ANC visits are still major hindrance to achieving prevention goals. Multiple pregnancies in 14–18 years old and women who had one to three pregnancies represent the other clusters affected by malaria. This study therefore suggests strengthening ANC strategies, while focusing on promoting the use of LLINs and SP, sexual education against early pregnancy, and counseling during first pregnancies might improve the current situation.

## Figures and Tables

**Table 1:** Univariate analysis for risk factors associated with placental malaria and peripheral malaria. *N* = 1000.

Variables	Levels	Peripheral malaria	Placental malaria
		Crude OR (95% CI)	Crude OR (95% CI)
Age	(14, 18)	Reference	Reference
	(18, 35)	0.97 (0.64–1.51)	0.98 (0.64–1.54)
	(35, 45)	0.39 (0.11–1.05)	0.31 (0.07–0.93)
Marital status	Unmarried	Reference	Reference
	Married	0.88 (0.59–1.33)	1.00 (0.66–1.55)
Preterm delivery	Term	Reference	Reference
	Preterm	0.89 (0.63–1.26)	0.98 (0.68–1.40)
Residence	Kankan	Reference	Reference
	Forécariah	1.84 (1.11–3.07)	1.79 (1.08–3.00)
	Guéckédou	2.40 (1.48–3.96)	2.24 (1.38–3.71)
	N'Zérékoré	0.88 (0.50–1.56)	0.65 (0.35–1.19)
Lived time	≤6 months	Reference	Reference
	≥6 months	0.61 (0.41–0.93)	0.58 (0.39–0.88)
Characteristic of residence	Urban	Reference	Reference
	Rural	1.69 (1.18–2.41)	1.60 (1.10–2.31)
Source of water	Drilling	Reference	Reference
	Well	1.90 (1.15–3.30)	2.32 (1.34–4.27)
	Rainwater	1.31 (0.75–2.39)	1.66 (0.91–3.19)
Education	University	Reference	Reference
	Any level	2.22 (0.87–7.51)	2.11 (0.83–7.15)
	Primary	2.29 (0.84–8.04)	1.91 (0.69–6.77)
	Secondary	1.23 (0.45–4.33)	1.12 (0.40–3.96)
Profession of parturient	Housewife	Reference	Reference
	Freelance	0.78 (0.53–1.14)	0.75 (0.50–1.10)
	Civil servant	0.55 (0.32–0.90)	0.59 (0.34–0.97)
Profession of head of household	Civil servant	Reference	Reference
	Farmer	1.67 (1.02–2.75)	2.19 (1.30–3.79)
	Freelance	1.20 (0.76–1.93)	1.62 (0.99–2.72)
	Unemployed	1.65 (0.76–3.38)	1.78 (0.77–3.88)
Status of household	Monogamous	Reference	Reference
	Polygamous	1.10 (0.75–1.62)	0.99 (0.66–1.48)
	Single	1.14 (0.72–1.78)	1.00 (0.62–1.57)
Backwater	No	Reference	Reference
	Yes	1.33 (0.92–1.90)	1.34 (0.92–1.93)
Garbage	Yes	Reference	Reference
	No	0.77 (0.55–1.08)	0.78 (0.55–1.10)
Means of transport for ANC	Own	Reference	Reference
	Foot	2.06 (1.26–3.51)	1.91 (1.17–3.27)
	Taxi	1.12 (0.62–2.05)	0.96 (0.53–1.78)
Distance from ANC health facility	Yes	Reference	Reference
	No	1.26 (0.76–2.03)	1.14 (0.66–1.86)
Regular use LLINs	Yes	Reference	Reference
	No	2.78 (1.95–4.01)	3.14 (2.17–4.61)
ANC visits number	Normal ANC	Reference	Reference
	Low ANC	1.84 (1.23–2.82)	1.74 (1.16–2.69)
Gravidity	Primigravid	Reference	Reference
	Paucigravid	1.17 (0.78–1.76)	1.25 (0.83–1.87)
	Multigravida	1.14 (0.75–1.73)	0.86 (0.54–1.34)
Parity	Primparous	Reference	Reference
	Pauciparous	0.41 (0.09–2.89)	0.42 (0.09–2.93)
	Multiparous	0.56 (0.12–3.93)	0.46 (0.10–3.23)
SP dose	≤2 doses	Reference	Reference
	≥3 doses	0.66 (0.45–0.95)	0.56 (0.37–0.83)
Other antimalarial drugs	Yes	Reference	Reference
	No	1.09 (0.77–1.55)	0.90 (0.63–1.28)
Medicinal plant	Yes	Reference	Reference
	No	1.52 (1.04–2.28)	1.37 (0.93–2.06)

**Table 2:** Logistic regression with peripheral malaria and clusters of sociodemographic and antenatal characteristics.

	OR		*p* value
Cluster 7 (irregular use of LLINs, ANC ≥ 4 in Forécariah)	Ref		
Cluster 3 (irregular use of LLINs in Kankan, N'Zérokoré)	1.99	0.56–12.68	0.365
Cluster 5 (irregular use of LLINs in Forecariah and Guéckédou, CPN < 4CPN)	11.81	3.44–74.26	0.001
Cluster 8 (irregular use of LLINs with ANC ≥ 4 in Guéckédou)	16.50	2.79–140.86	0.004
Cluster 12 (regular use of LLINs, multigravida, age (14–18))	12.37	1.62–120.28	0.017
Cluster 13 (regular use of LLINs, multigravida, age (19–45))	1.03	0.24–7.05	0.970
Cluster 14 (regular use of LLINs, primigravids or paucigravids)	4.71	1.21–31.29	0.049
Cluster 15 (regular use of LLINs, paucigravids)	1.46	0.41–9.31	0.616

**Table 3:** Logistic regression with placental parasitemia and clusters of sociodemographic and antenatal characteristics.

Clusters	OR	95% CI	*p* value
Cluster 11 (regular use of LLINs, multigravida)	Ref		
Cluster 5 (ANC ≥ 4, irregular use of LLINs in Forécariah)	1.18	0.17–5.14	*p*=0.843
Cluster 6 (ANC < 4, irregular use of LLINs in Forécariah)	10.12	4.54–25.84	*p* < 0.001
Cluster 7 (irregular use of LLINs in Kankan and N'Zérokoré)	2.24	0.99–5.74	*p*=0.068
Cluster 8 (irregular use of LLINs in Guéckedou)	19.94	8.73–51.99	*p* < 0.001
Cluster 12 (regular use of LLINs, primigravids and paucigravids)	5.05	1.95–14.12	*p*=0.001
Cluster 13 (regular use of LLINs, pauciparous)	1.58	0.70–4.04	*p*=0.297

## Data Availability

Datasets in Commas Separated Value (csv) format and R script used to support the findings are available from corresponding author upon request.

## Supplementary Materials

Supplementary Materials Supplementary file 1: description of study sample. Supplementary file 2: relation age and marital status. Supplementary file 3, Table 1: Univariate analysis using both peripheral and placental infection as dependant variables. Supplementary file 3, Table 2: univariate analysis using only peripheral infection as dependant variable.
