# Supplementary material for: Malaria-Associated Factors among Pregnant Women in Guinea
Source: J Trop Med. 2019 Nov 15;2019:3925094. doi: 10.1155/2019/3925094 (PMC6925697; doi:10.1155/2019/3925094)
Supplement: Supplementary Materials — Supplementary file 1: description of study sample. Supplementary file 2: relation age and marital status. Supplementary file 3, Table 1: Univariate analysis using both peripheral and placental infection as dependant variables. Supplementary file 3, Table 2: univariate analysis using only peripheral infection as dependant variable. [file 3925094.f1.zip › 3925094.f1/Additionnal file 1.pdf]

Table : Characteristics of study population (N=1000)

Demographic characteristics	n(%)
Age (years)	
[14,18]	193 (19.3)
(18,35]	752 (75.2)
(35,45]	55 (5.5)
Marital status	
unmarried	209 (20.9)
married	791 (79.1)
Preterm delivery	
No	604 (60.4)
Yes	396 (39.6)
Residence	
Kankan	250 (25.0)
Forécariah	250 (25.0)
Guéckédou	250 (25.0)
N’zérokoré	250 (25.0)
Lived time	
<6 months	180 (18.0)
> 6 months	820 (82.0)
Characteristic of residence	
Urban	727 (72.7)
Rural	273 (27.3)
Source of water	
Drilling	176 (17.6)
Well	518 (51.8)
Rainwater	306 (30.6)
Level of education	
Any level	562 (56.2)
Primary	159 (15.9)
Secondary	234 (23.4)
University	45 (4.5)
Profession of parturients	

Housewife	469 (46.9)
Freelance	348 (34.8)
Civil servant	183 (18.3)
Head of household profession	
Civil servant	236 (23.6)
Farmer	251 (25.1)
Freelance	451 (45.1)
Unemployed	62 (6.2)
Status of household	
monogamous	492 (49.2)
polygamous	312 (31.2)
single	196 (19.6)
Stagnant water	
No	709 (70.9)
Yes	291 (29.1)
Garbage	
Yes	420 (42.0)
No	580 (58.0)
Means of transport	
Owner	188 (18.8)
Feet	539 (53.9)
Taxi	273 (27.3)
Distance to ANC health facility	
Yes	877 (87.7)
No	123 (12.3)
Regular use of MILDA	
Yes	538 (53.8)
No	462 (46.2)
ANCnumber	
Normal ANC	300 (30.0)
Low ANC	700 (70.0)
Gravidity	

Primigravid	376 (37.6)	
Paucigravid	330 (33.0)	
Multigravid	294 (29.4)	
Parity		
Primiparous	7 (0.7)	
Pauciparous	597 (59.7)	
Multiparous	396 (39.6)	
SP doses		
≤2 doses	645 (64.5)	
≥3 doses	355 (35.5)	
Other antimalarial drugs		
Yes	403 (40.3)	
No	597 (59.7)	
Continued		
Medicinal plant		
Yes	312 (31.2)	
No	688 (68.8)	
Newborn		
Living	945 (94.5)	
Dead	55 (5.5)	
Peripheral malaria		
Negative	842 (84.2)	
Positive	158 (15.8)	
Placental malaria		
Negative	852 (85.2)	
Positive	148 (14.8)	
Newborn malaria		
Negative	972 (97.2)	
Positive	28 (2.8)	
Weight		
Normal	887 (88.7)	
LBW	113 (11.3)	
**Other characteristics**		**means (sd))**
Age		24.38±6.2
Peripheral Density		1872.30 (12317.16)

Placental density	1478.64 (9665.67)
Density for newborn	2.90 (33.37)
Temperature parturients	37.07 (0.60)
Temperature newborns	36.39 (0.64)
APGAR1	8.68 (2.13)
APGAR2	9.32 (2.29)
