# Supplementary material for: Malaria-Associated Factors among Pregnant Women in Guinea
Source: J Trop Med. 2019 Nov 15;2019:3925094. doi: 10.1155/2019/3925094 (PMC6925697; doi:10.1155/2019/3925094)
Supplement: Supplementary Materials — Supplementary file 1: description of study sample. Supplementary file 2: relation age and marital status. Supplementary file 3, Table 1: Univariate analysis using both peripheral and placental infection as dependant variables. Supplementary file 3, Table 2: univariate analysis using only peripheral infection as dependant variable. [file 3925094.f1.zip › 3925094.f1/Additionnal file 3.pdf]

Table 1: univariate analysis using women who had both peripheral and placental malaria. N=1000

Variables	Levels	No (%)	Yes (%)	OR	95% CI
Age	[14,18]	167 (19.0)	26 (21.3)		
	(18,35]	657 (74.8)	95 (77.9)	0.93	0.59-1.51
	(35,45]	54 (6.2)	1 (0.8)	0.12	0.01-0.58
Marital status	Unmarried	182 (20.7)	27 (22.1)		
	Married	696 (79.3)	95 (77.9)	0.92	0.59-1.48
Preterm delivery	Term	527 (60.0)	77 (63.1)		
	Preterm	351 (40.0)	45 (36.9)	0.88	0.59-1.29
Residence	Kankan	228 (26.0)	22 (18.0)		
	Forecariah	207 (23.6)	43 (35.2)	2.15	1.26-3.78
	Gueckédou	204 (23.2)	46 (37.7)	2.34	1.37-4.08
	N'Zérékoré	239 (27.2)	11 (9.0)	0.48	0.22-0.99
Lived time	≤6 months	146 (16.6)	34 (27.9)		
	≥6 months	732 (83.4)	88 (72.1)	0.52	0.34-0.80
Characteristic of residence	Urban	650 (74.0)	77 (63.1)		
	Rural	228 (26.0)	45 (36.9)	1.67	1.11-2.47
Source of water	Drilling	164 (18.7)	12 (9.8)		
	Well	442 (50.3)	76 (62.3)	2.35	1.29-4.65
	Rainwater	272 (31.0)	34 (27.9)	1.71	0.88-3.52
Education	University	43 (4.9)	2 (1.6)		
	Illeterate	483 (55.0)	79 (64.8)	3.52	1.05-21.83
	Primary	138 (15.7)	21 (17.2)	3.27	0.91-20.97
	Secondary	214 (24.4)	20 (16.4)	2.01	0.56-12.88
Profession of parturient	Housewife	402 (45.8)	67 (54.9)		
	Freelance	310 (35.3)	38 (31.1)	0.74	0.48-1.12
	Civil servant	166 (18.9)	17 (13.9)	0.61	0.34-1.05
Profession of head of household	Civil servant	217 (24.7)	19 (15.6)		
	Farmer	211 (24.0)	40 (32.8)	2.17	1.23-3.93
Continued					
	Freelance	397 (45.2)	54 (44.3)	1.55	0.91-2.75

	Unemployed	53 (6.0)	9 (7.4)	1.94	0.80-4.43
Status of household	Monogamous	437 (49.8)	55 (45.1)		
	polygamous	271 (30.9)	41 (33.6)	1.20	0.78-1.85
	Single	170 (19.4)	26 (21.3)	1.22	0.73-1.98
Backwater	No	631 (71.9)	78 (63.9)		
	Yes	247 (28.1)	44 (36.1)	1.44	0.96-2.14
Garbage	No	365 (41.6)	55 (45.1)		
	Yes	513 (58.4)	67 (54.9)	0.87	0.59-1.27
Means of transport for ANC	Owner	176 (20.0)	12 (9.8)		
	Feet	451 (51.4)	88 (72.1)	2.86	1.59-5.63
	Taxi	251 (28.6)	22 (18.0)	1.29	0.63-2.75
Distance from ANC health facility	Yes	773 (88.0)	104 (85.2)		
	No	105 (12.0)	18 (14.8)	1.27	0.72-2.14
Regular use LLINs	Yes	506 (57.6)	32 (26.2)		
	No	372 (42.4)	90 (73.8)	3.83	2.53-5.93
ANC visits number	Normal ANC	276 (31.4)	24 (19.7)		
	Low ANC	602 (68.6)	98 (80.3)	1.87	1.19-3.05
Gravidity	Primigravid	331 (37.7)	45 (36.9)		
	Paucigravid	285 (32.5)	45 (36.9)	1.16	0.75-1.81
	Multigravida	262 (29.8)	32 (26.2)	0.90	0.55-1.45
Parity	Primparous	5 (0.6)	2 (1.6)		
	Pauciparous	530 (60.4)	67 (54.9)	0.32	0.07-2.24
	Multiparous	343 (39.1)	53 (43.4)	0.39	0.08-2.74
SP dose	≤2 doses	553 (63.0)	92 (75.4)		
	≥3 doses	325 (37.0)	30 (24.6)	0.55	0.35-0.85
Other antimalarial drugs	Yes	354 (40.3)	49 (40.2)		
	No	524 (59.7)	73 (59.8)	1.01	0.69-1.49
Medicinal plant	Yes	282 (32.1)	30 (24.6)		
	No	596 (67.9)	92 (75.4)	1.45	0.95-2.28

Table 2: Univariate analysis using women who had only peripheral malaria.N=36

Variables	Levels	No (%)	Yes (%)	OR	95% CI
Age	[14,18]	187 (19.4)	6 (16.7)		
	(18,35]	725 (75.2)	27 (75.0)	1.16	0.50-3.15
	(35,45]	52 (5.4)	3 (8.3)	1.80	0.37-7.06
Marital status	Unmarried	200 (20.7)	9 (25.0)		
	Married	764 (79.3)	27 (75.0)	0.79	0.38-1.79
Preterm delivery	Term	582 (60.4)	22 (61.1)		
	Preterm	382 (39.6)	14 (38.9)	0.97	0.48-1.90
Residence	Kankan	244 (25.3)	6 (16.7)		
	Forecariah	246 (25.5)	4 (11.1)	0.66	0.17-2.34
	Gueckédou	238 (24.7)	12 (33.3)	2.05	0.78-5.97
	N'Zérékoré	236 (24.5)	14 (38.9)	2.41	0.95-6.91
Lived time	≤6 months	175 (18.2)	5 (13.9)		
	≥6 months	789 (81.8)	31 (86.1)	1.38	0.57-4.07
Characteristic of residence	Urban	704 (73.0)	23 (63.9)		
	Rural	260 (27.0)	13 (36.1)	1.53	0.74-3.02
Source of water	Drilling	169 (17.5)	7 (19.4)		
	Well	497 (51.6)	21 (58.3)	1.02	0.45-2.63
	Rainwater	298 (30.9)	8 (22.2)	0.65	0.23-1.88
Education	University	43 (4.5)	2 (5.6)		
	Illeterate	541 (56.1)	21 (58.3)	0.83	0.23-5.33
	Primary	151 (15.7)	8 (22.2)	1.14	0.27-7.73
	Secondary	229 (23.8)	5 (13.9)	0.47	0.10-3.35
Profession of parturient	Housewife	450 (46.7)	19 (52.8)		
	Freelance	334 (34.6)	14 (38.9)	0.99	0.48-2.00
	Civil servant	180 (18.7)	3 (8.3)	0.39	0.09-1.18
Profession of head of household	Civil servant	225 (23.3)	11 (30.6)		
	Farmer	242 (25.1)	9 (25.0)	0.76	0.30-1.87
	Freelance	438 (45.4)	13 (36.1)	0.61	0.27-1.40
	Unemployed	59 (6.1)	3 (8.3)	1.04	0.23-3.46
Status of household	Monogamous	473 (49.1)	19 (52.8)		
	polygamous	302 (31.3)	10 (27.8)	0.82	0.36-1.76
	Single	189 (19.6)	7 (19.4)	0.92	0.36-2.14
Backwater	No	683 (70.9)	26 (72.2)		
	Yes	281 (29.1)	10 (27.8)	0.93	0.42-1.91
Garbage	No	400 (41.5)	20 (55.6)		
	Yes	564 (58.5)	16 (44.4)	0.57	0.29-1.11
Means of transport for ANC	Owner	180 (18.7)	8 (22.2)		
	Feet	521 (54.0)	18 (50.0)	0.78	0.34-1.92
	Taxi	263 (27.3)	10 (27.8)	0.86	0.33-2.28
Distance from ANC health facility	Yes	846 (87.8)	31 (86.1)		
	No	118 (12.2)	5 (13.9)	1.16	0.39-2.79

Regular use LLINs	Yes	518 (53.7)	20 (55.6)		
	No	446 (46.3)	16 (44.4)	0.93	0.47-1.81
ANC visits number	Normal ANC	292 (30.3)	8 (22.2)		
	Low ANC	672 (69.7)	28 (77.8)	1.52	0.72-3.61
Gravidity	Primigravid	366 (38.0)	10 (27.8)		
	Paucigravid	320 (33.2)	10 (27.8)	1.14	0.46-2.82
	Multigravida	278 (28.8)	16 (44.4)	2.11	0.95-4.87
Parity	Primparous	7 (0.7)	0 (0.0)		
	Pauciparous	580 (60.2)	17 (47.2)	458746.	74 (0.00-NA
	Multiparous	377 (39.1)	19 (52.8)	788795.	31 (0.00-NA
SP dose	≤2 doses	623 (64.6)	22 (61.1)		
	≥3 doses	341 (35.4)	14 (38.9)	1.16	0.57-2.28
Other antimalarial drugs	Yes	391 (40.6)	12 (33.3)		
	No	573 (59.4)	24 (66.7)	1.36	0.69-2.85
Medicinal plant	Yes	304 (31.5)	8 (22.2)		
	No	660 (68.5)	28 (77.8)	1.61	0.76-3.83
