# Supplementary figures and images for: Malaria-Associated Factors among Pregnant Women in Guinea
Source: J Trop Med. 2019 Nov 15;2019:3925094. doi: 10.1155/2019/3925094 (PMC6925697; doi:10.1155/2019/3925094)

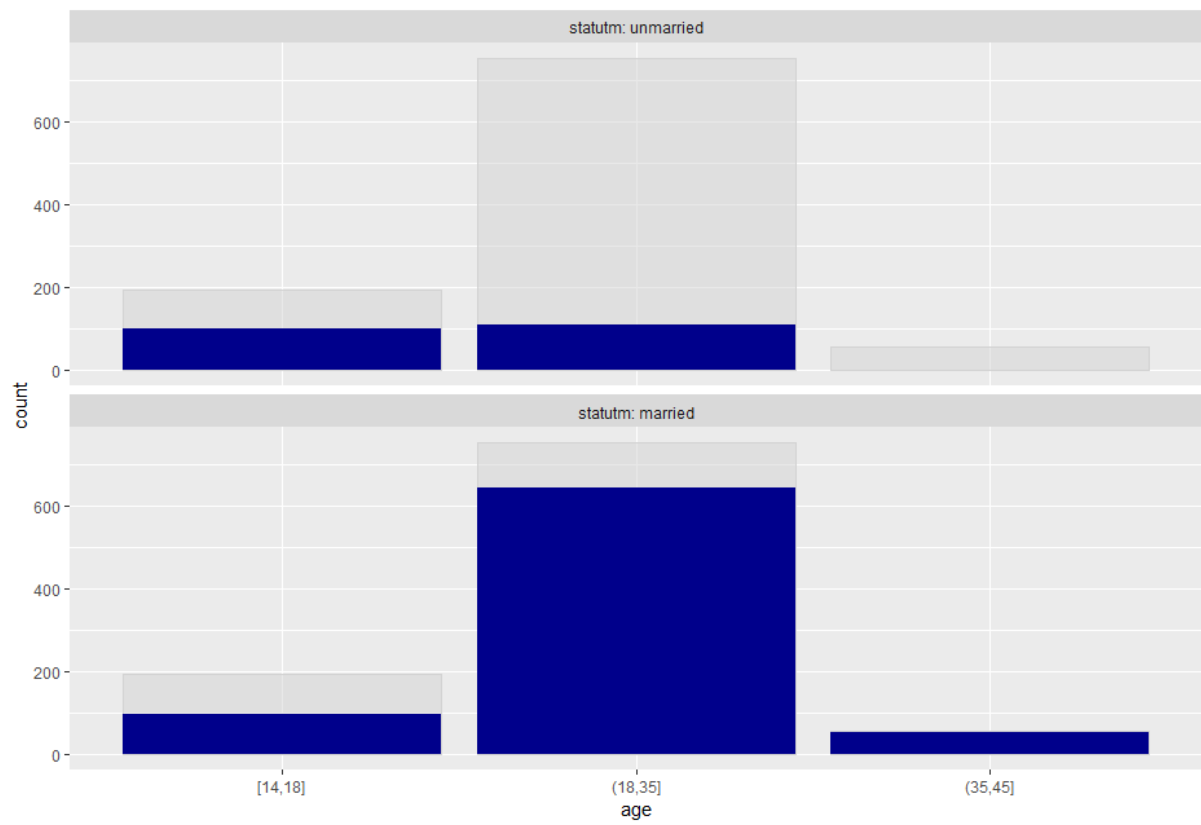

Figure 3: relation marital status and age

Supplement: Supplementary Materials — Supplementary file 1: description of study sample. Supplementary file 2: relation age and marital status. Supplementary file 3, Table 1: Univariate analysis using both peripheral and placental infection as dependant variables. Supplementary file 3, Table 2: univariate analysis using only peripheral infection as dependant variable. [file 3925094.f1.zip › 3925094.f1/Additionnal file 2.pdf]
